# Fuzzy Edge-Detection as a Preprocessing Layer in Deep Neural Networks for Guitar Classification

**DOI:** 10.3390/s22155892

**Published:** 2022-08-07

**Authors:** Cesar Torres, Claudia I. Gonzalez, Gabriela E. Martinez

**Affiliations:** Tijuana Institute of Technology/TECNM, Tijuana 22414, Mexico

**Keywords:** convolutional neural networks, guitar recognition, fuzzy edge-detection

## Abstract

Deep neural networks have demonstrated the capability of solving classification problems using hierarchical models, and fuzzy image preprocessing has proven to be efficient in handling uncertainty found in images. This paper presents the combination of fuzzy image edge-detection and the usage of a convolutional neural network for a computer vision system to classify guitar types according to their body model. The focus of this investigation is to compare the effects of performing image-preprocessing techniques on raw data (non-normalized images) with different fuzzy edge-detection methods, specifically fuzzy Sobel, fuzzy Prewitt, and fuzzy morphological gradient, before feeding the images into a convolutional neural network to perform a classification task. We propose and compare two convolutional neural network architectures to solve the task. Fuzzy edge-detection techniques are compared against their classical counterparts (Sobel, Prewitt, and morphological gradient edge-detection) and with grayscale and color images in the RGB color space. The fuzzy preprocessing methodologies highlight the most essential features of each image, achieving favorable results when compared to the classical preprocessing methodologies and against a pre-trained model with both proposed models, as well as achieving a reduction in training times of more than 20% compared to RGB images.

## 1. Introduction

Artificial intelligence (AI) allows us to automate multiple tasks in different areas, such as artificial vision, which makes it possible to perform several jobs that were previously believed only humans would be capable of doing. The tasks are those such as object identification or object location in digital images; processes such as these require previous knowledge of the objects you are looking for, as well as the context in which they are found, requirements that are completely related to the vision and cognitive abilities of humans.

In recent years, deep neural networks (DNN) have made a huge advances in recognition tasks in multiple areas due to the capabilities of feature-extraction layers that DNNs have embedded in their design, which make them very attractive in multiple disciplines. Zhang et al. [1] created a fruit classification system using a deep neural network to replace handcrafted features, beating state-of-the-art approaches. Horng et al. [2] used a DNN, feeding it with aerial images to classify tree areas and help in the understanding of land use. Sebti et al. [3] provided a solution for forecasting and diagnosing of diabetic retinopathy by training a convolutional neural network with retina images, achieving over 96% accuracy.

Previous authors tackled the guitar/instrument classification problem, for example, Rabelo et al. [4] used a support vector machine to classify the sounds of guitars (in a binary problem) from two brands, obtaining 95% accuracy with a particular approach. Yet, this was done by using the sound (music notes) generated by the instrument, instead of images, to perform the classification tasks. Banerjee et al. [5] used musical notes and support vector machines to classify string instruments (cello, double bass, guitar, and violin), obtaining up to 100% recognition with random forest. This paper proposes an artificial vision system that performs the classification by body types of some of the most popular guitar styles found nowadays. The system operates by exploiting the capabilities of the feature-extraction layers of DNN, feeding them with pictures of the guitars that need to be classified by the user. Given the lack of previous attempts to do so, we created a dataset by scraping images from popular search engines to generate a competent database that contained sufficient images of some of the most popular guitar models. The application of image-based instrument-classification systems could have great relevance in retail stores and warehouses of music instrument franchises, where there is a constant flow of inventory involved, especially of used products. Intelligent systems could be applied for inventory keeping or organization of unlabeled items. Another form of implementation viable for the music industry is a complementary system for off-site quoting services for instrument purchases or store credit requests, making it more compelling for customers to sell used instruments to the store for resale.

The process of detecting edges is an essential part of pattern-recognition systems; it simplifies the analysis by reducing the image to its minimal expression, and by doing so, it reduces the amount of processing needed [6]. This could be considered a difficult task, especially when the images contain noise or include irrelevant information. To solve this challenge, some fuzzy edge-detectors have been proposed. In Tao et al. [7], Hu et al. [8], and Ontiveros-Robles et al. [9], the authors presented some edge-detection methodologies with their foundation in type-1 fuzzy systems, T1FS. In Mendoza et al. [10,11], the authors propose fuzzy edge-detection based on the Sobel operator and interval type-2 fuzzy systems IT2FS. In Biswas et al. [12] and Melin et al. [13], the Canny method and the morphological gradient approach were improved using IT2FS, respectively. In the area of pattern-recognition, fuzzy edge-detection methods play an important role in improving the recognition rate when comparing the results with images without processing or when traditional filters are applied. We can mention other research works, where some edge-detection methodologies were based on T1FS, IT2FS, and general type-2 fuzzy sets (GT2FS) used in the preprocessing pipeline for face-recognition based on a monolithic neural network [14,15]. Mendoza et al. [16] applied edge-detectors to two face databases; this detection system was based on T1 and IT2FS. The edges found were used as inputs for a face-recognition system; the authors concluded that the recognition achieved by the system was improved when fuzzy edge-detection methods were applied. Martinez et al. [17] presented a face-recognition method with its foundation in modular neural networks, with favorable results when the fuzzy Sobel edge-detection was performed.

The success of convolutional neural networks (CNNs) in classification is due to their ability to perform both feature-extraction and classification, and most models perform very well without preprocessing steps. However, sometimes, the dataset images are unbalanced, have lower resolution, poor quality, or acquired some noise or uncertainty during the capture process. Due to these facts, some approaches use additional preprocessing methods, including image resizing, data augmentation, cropping, converting to grayscale to reduce the preprocessing time, and adding filters and equalization to improve the image quality or resolution. In the literature, we can find some works that have shown that the use of preprocessing methods before CNN-based models improves the results. Cordero-Martínez et al. [18,19] presented a comparison of four image preprocessing methods to classify diabetic retinopathy using CNN, and the authors evidenced and concluded that the preprocessing steps are important to increase the accuracy of the results. Kato et al. [20] proposed a preprocessing approach applied before a CNN-based model, and the results were 34.8% higher than the conventional CNN model. In Musallam et al. [21], three preprocessing steps were proposed to enhance the quality of MRI (magnetic resonance imaging) before these were introduced to a deep convolutional neural network (DCNN), which involved removing confusing objects, using a non-local mean algorithm (NLM), and applying histogram equalization. These were applied for automatic detection of brain tumors in MRI images and experimental results proved the robustness of the proposed architecture, which increased the detection accuracy of a variety of brain diseases in a short time compared to other well-known deep convolutional neural network (DCNN) models such as VGG16 [22], VGG19 [22], and hybrid CNN-SVM [23]. Finally, Lăzărescu et al. [24] presented an algorithm for fingerprint classification using a combination of edge features and CNN. In this work, some preprocessing steps were applied, including edge-enhancement operations, data resizing, data augmentation, and the images were enhanced using Prewitt and Laplacian of Gaussian. The proposed algorithm achieved a very good performance compared to the traditional hand-crafted features.

The motivation to implement fuzzy edge-detection in this paper as a preprocessing phase was that fuzzy logic is a good technique to model the uncertainty or noise encountered in images where, with an appropriate filtering operator, this will be suppressed. The fuzzy methodologies (Prewitt, Sobel, and morphological gradient fuzzy edge-detectors) presented in this paper consider implementing only T1 fuzzy sets and are referenced from the previous state-of-the-art research [25,26]. The implementation and combination of fuzzy preprocessing techniques and convolutional neural networks produce powerful artificial intelligence tools for pattern recognition.

## 2. Materials and Methods

### 2.1. Theoretical Concepts

#### 2.1.1. Convolutional Neural Network

The convolutional neural network, also known as CNN, is one of the most popular deep-learning techniques. where a computer system imitates the visual cortex and brain to identify patterns from visual elements [27]. CNNs have been used in multiple fields, such as medical [28,29,30,31], autonomous vehicles [32,33,34], and agricultural [35,36], just to name a few. A CNN differs from a conventional neural network (NN) because it contains at least a convolutional layer. The basic architecture of a CNN consists of the following layers: input, convolutional, activation fn., pooling, dense, and output layer.

1. Input layer: The input layer constitutes the input of the data that are going to be fed to the network, with a fixed size;
2. Convolutional layer: This layer applies a scalar product with a small matrix (kernel) to extract features or patterns from the input. generating a new matrix with the extracted contents;
3. Activation function: CNNs use activation functions to trigger the neurons when the individual values reach a threshold; these functions allow us to normalize the input data and perform non-linearization of the data. One of the most used activations is a rectified linear unit (ReLu); the output of the activation of a ReLu is the maximum between the values of the input;
4. Pooling layer: This layer allows us to create a smaller representation of the input data, and by doing so, we can reduce the number of features held by the input. The maximum pooling layer is one of the most used layers; this layer uses a filter with size n × n, which does a pass all over the input, as the convolution layer. In this case, the maximum value of this filter is stored as the output in the new representation, reducing the dimensions of the input proportionally to the dimensions of the filter and its stride;
5. Dense layer (fully connected): Consists of the weights and biases (like a traditional feed-forward neural network). The input of these layers is the output of a convolution or pooling layer;
6. The output layer: This layer contains the neurons that will provide the output of the input that was fed through the model.

#### 2.1.2. Fuzzy Logic Systems

Fuzzy logic aims to perform representations in nonlinear ways as human logic does. Often, linguistic terms are used that differ from conventional logic where it is usually binary, and fuzzy logic allows gradual representations in a continuous space, thereby allowing levels of uncertainty. Zadeh introduced the fuzzy sets given the discontent of classic sets (crisp sets) [37]. Fuzzy sets allow the use of membership, meaning their elements are capable of being part of one or more classes at the same time. The range of these sets is defined as human logic, where they depend on the concept or user applying them.

The T1FS set A is from Universe X that goes from [0, 1], belonging to a continuous function, i.e., $\mu_{A}:X\to \left[0,1\right]$. The T1 membership function *A* is denoted as $\mu_{A}\left(x\right);$ this function is defined in Equation (1).

(1) $$A=\{\left(x,\;\mu_{A}\left(x\right)\right)|x\in X\;\}$$

The different membership functions (MFs) most used to represent a fuzzy set are the following: the triangular MF, the trapezoidal MF, and the Gaussian MF, which is used in the fuzzy edge-detection approach presented in this paper. This last consists of two parameters {c, σ} and is expressed in Equation (2); variable c represents the mean of the MF and σ the amplitude.

(2) $$gaussian\left(x;c,\sigma \right)=exp-\frac{1}{2}{\left(\frac{x-c}{\sigma }\right)}^{2}$$

A fuzzy inference system (FIS) is based on if-then rules with fuzzy reasoning [38] applied to fuzzy sets. The basic structure of a FIS constitutes a database, along with a reasoning mechanism, which infers a reasonable conclusion from the inputs, outputs, and the knowledge provided by the database. Popular fuzzy inference systems are: Mamdani [39], Tsukamoto, and Takagi-Sugeno-Kang [40].

#### 2.1.3. Edge-Detection Methods

Image-processing techniques consist of the manipulation of digital images to hide or highlight details, specific patterns, improve illumination, or eliminate noise caused by external agents such as artifacts that can be caused by the camera sensor or movement when taking the picture. This processing consists of applying an operation in a pixel window (kernel), which goes through the images, changing their content uniformly to create a new image [41]. Equation (3) describes the process of the application of a kernel to an image, where the k represents the kernel, r,c represents the coordinates of the value of the kernel, where r represents the row and c the column, and the variable f represents the input image.

(3) $$g\left(x,y\right)=\overset{a}{\underset{r=-a}{\sum }}\overset{b}{\underset{c=-b}{\sum }}k\left(r,c\right)f\left(x+r,y+c\right)$$

Operations such as edge-detection are performed to reduce the amount of information that an image contains. Edge-detection techniques identify discontinuities in the brightness levels of the image to identify borders.

Among the most popular edge-detection techniques are the Roberts, Sobel, and Prewitt operators [42,43,44], which are focused on calculating the gradient with the first derivate of an image by applying a convolution operation that approximates the gradient and returns the first derivate for the horizontal and vertical directions.

##### Prewitt and Sobel Edge-Detection Methods

The traditional Prewitt and Sobel operators work in almost the same way; they both consists of a 3×3  neighborhood gradient operator, but they differ in the mask used in the convolutional process. The masks of the convoluted Prewitt operator on a grayscale image are defined by Equations (4) and (5), which correspond to ${\mathrm{Prewittx}}_{\;}$ and Prewitty, respectively. In contrast, the masks used in the Sobel operator are expresed in Equation (6) (Sobelx) and Equation (7) (Sobely).

(4) $$Prewittx=\left[\begin{array}{ccc} -1 & -1 & -1\\ 0 & 0 & 0\\ 1 & 1 & 1 \end{array}\right]\;$$

(5) $$Prewitty=\left[\begin{array}{ccc} -1 & 0 & 1\\ -1 & 0 & 1\\ -1 & 0 & 1 \end{array}\right]\;$$

(6) $$Sobelx=\left[\begin{array}{ccc} -1 & -2 & -1\\ 0 & 0 & 0\\ 1 & 2 & 1 \end{array}\right]\;$$

(7) $$Sobely=\left[\begin{array}{ccc} -1 & 0 & 1\\ -2 & 0 & 2\\ -1 & 0 & 1 \end{array}\right]\;$$

**Figure 1:**
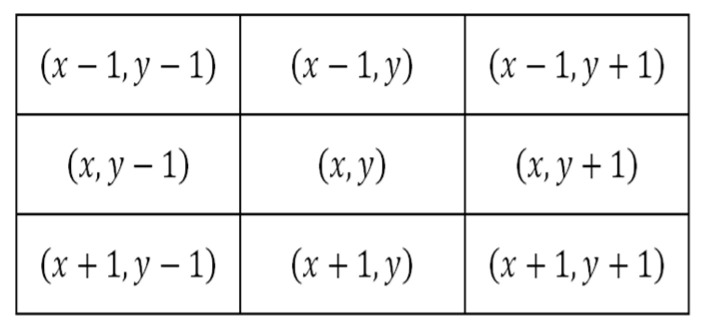
Coordinates of the input image *f*.

The filter applies two different kernels to an image to generate the gradients *g_x_* in Equation (8) for a horizontal orientation and *g_y_* in Equation (9) for a vertical orientation. The coordinates of the input f are demonstrated in Figure 1, where the axis is represented by *x* for the horizontal axis and *y* for the vertical axis, and *f* for the image (input) source [26]. In Equations (7) and (8), kernelx represents the mask Prewittx or Sobelx, and kernely is the mask for Prewittx or Sobely, depending on the filter to be calculated.

(8) $$g_{x}=\overset{i=3}{\underset{i=1}{\sum }}\overset{j=3}{\underset{j=1}{\sum }}kernelx_{i,j\;}*\;f_{x+i-2,\;y+j-2}$$

(9) $$g_{y}=\overset{i=3}{\underset{i=1}{\sum }}\overset{j=3}{\underset{j=1}{\sum }}kernely_{i,j\;}*\;f_{x+i-2,\;y+j-2}$$

The magnitude of the gradients is obtained with Equation (10), which takes into consideration the results of the calculations of *g_x_* and *g_y_* from the image f using Equations (8) and (9); these convolutions are applied using the kernels defined in Equations (4)–(7).

(10) $$G\left[f\left(x,y\right)\right]=\sqrt{g_{x}^{2}+g_{y}^{2}}$$

##### Morphological Gradient (MG)

**Figure 2:**
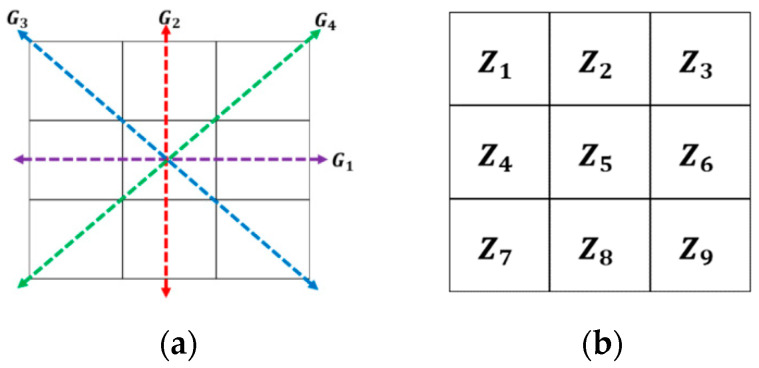
(**a**) Gradient directions $G_{i}$. (**b**) Coefficients $Z_{i}$.

MG is an edge-detection technique that calculates the first derivate of an image in the four orientations of the image, i.e., vertical, horizontal, and its diagonals (0°,45°,90°, and 135°). This is demonstrated in Figure 2a, where the gradients are indicated by the following variables *G*_1_, *G*_2_, *G*_3_, and *G*_4_. The calculation of the gradients is made in the following way.

*G_i_* (where *I* = 1…4) represents the direction of the edge (gradient). It is calculated with Equation (11) by using a 3 × 3 kernel. In Equation (11), *z_i_* represent the coefficient of each of the matrix positions shown in Figure 2b, where the coefficients are calculated with Equation (12); *f* is the representation of the image, i.e., the x-axis for the columns, and y-axis for the rows. The edge value is denoted with the variable “Edges”, which is integrated using Equation (13) [13,25,45].

(11) $$G_{1}=\sqrt{{\left(z_{5\;}-z_{2}\right)}^{2\;}+{\left(z_{5\;}-z_{8}\right)}^{2\;}},G_{2}=\sqrt{{\left(z_{5\;}-z_{4}\right)}^{2\;}+{\left(z_{5\;}-z_{6}\right)}^{2\;}},G_{3}=\;\sqrt{{\left(z_{5\;}-z_{1}\right)}^{2\;}+{\left(z_{5\;}-z_{9}\right)}^{2\;}},G_{4}=\sqrt{{\left(z_{5\;}-z_{3}\right)}^{2\;}+{\left(z_{5\;}-z_{7}\right)}^{2\;}}\;$$

(12) $$z_{1}=f\left(x-1,y-1\right),\;z_{2\;}=f\left(x,y-1\right),\;z_{3}=f\left(x+1,\;y-1\right),\;z_{4}=f\left(x-1,y\right),\;\;z_{5\;}=f\left(x,y\right),\;\;z_{6}=f\left(x+1,\;y\right),\;z_{7}=f\left(x-1,y+1\right),\;z_{8\;}=f\left(x,y+1\right),\;z_{9}=f\left(x+1,\;y+1\right)$$

(13) $$Edges=G_{1}+G_{2}+G_{3}+G_{4}$$

### 2.2. Methodology

**Figure 3:**
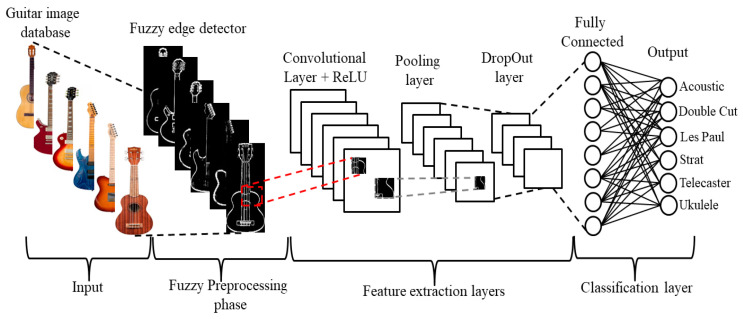
General approach.

The purpose of this investigation was to compare the effect of performing diverse image preprocessing techniques before feeding the images into a CNN to perform a classification test. Among the techniques performed were fuzzy edge-detection filters. Fuzzy filters (in this case edge-detection filters) allow us to determine the membership of a pixel to a boundary or a uniform region of the image, taking in consideration uncertainty. The general methodology of the proposal is illustrated in Figure 3 and explained as follows.

#### 2.2.1. Input Database

In this study case, we used a self-made dataset that contained a balanced distribution of images of six different guitar styles (acoustic guitar, double cut, Les Paul, telecaster, stratocaster, and ukulele). Each class had a high degree of similarity with another, as denoted by the following pairings: acoustic with ukulele, double cut with Les Paul, and telecaster with stratocaster. These similarities only applied to our object of interest, while the images contained high levels of variation between each other. Since the images were scraped from the web by using popular search engines, the results included stock images for product advertisements from popular brands [46,47,48], or pictures posted in forums by owners of the instruments. The variations included a difference in position, orientation, and rotation of the object, illumination, other objects present in the frame (hands, cases, stands, straps, or multiple guitars in the background), and resolution. The guitar database created consisted of 5400 images in color format (see Table 1), with the classes balanced (900 images per class), and with normalized dimensions of 150 × 150 pixels. The process of creating the dataset was as follows:

1. Create a list of keywords to perform a search in the search engines (Google Images and Bing Images); some of the keywords used are the follows:
  - Example of keywords used for the Les Paul class: “Gibson Les Paul, Les Paul style guitar, LP Junior, Les Paul Studio, Epiphone Les Paul, Les Paul, Les Paul copy, Ibanez Les Paul body guitar, ESP Les Paul, Burny Les Paul”;
  - Example of keywords used for Telecaster class: “Fender telecaster, Squier Tele, telecaster, telecaster type guitar, telecaster style guitar, tele guitar, telecaster guitar, Fender telecaster USA, telecaster custom shop”;
2. Download the top 100 hits from each keyword and save the images with a fixed size (150 × 150 px);
3. Manually prune the dataset to remove duplicates or outliers.

#### 2.2.2. Fuzzy Preprocessing Phase

This section contains an explanation of the membership function used, as well as the fuzzy rules and the methodology for the implementation of the fuzzy edge-detection approaches.

##### Fuzzy Sobel and Fuzzy Sobel Edge-Detection

The process to generate the fuzzy Sobel and fuzzy Prewitt edge-detection methods is similar, depending on the operator to be applied, only changing the mask, as previously explained in Section 2.1.3 and expressed with Equations (4)–(7). The inference system is a type-1 fuzzy Mamdani FIS with two inputs, one output, and three fuzzy rules. The general methodology is explained as follows, emphasizing that the numerical results used as an example are calculated with fuzzy Sobel.

**Figure 4:**
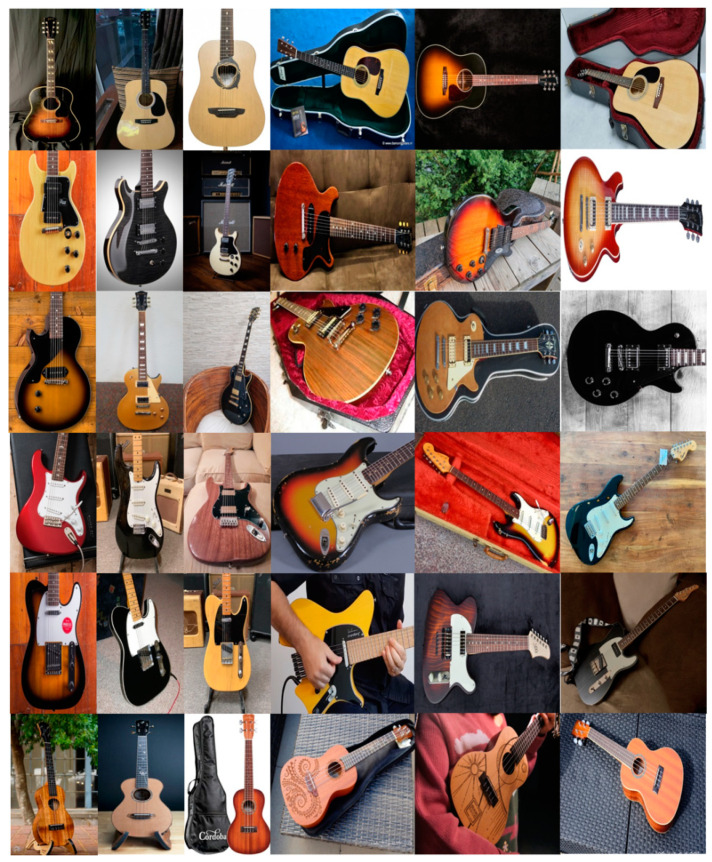
Sample of the guitar database.


**Figure 5:**
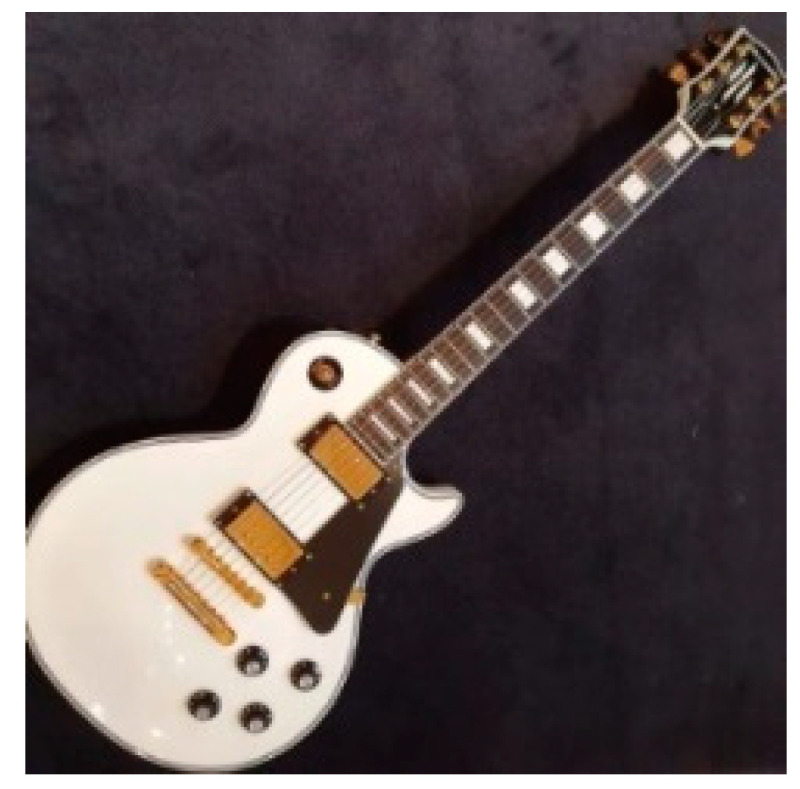
Input guitar image.

1. Read the input image. First, we need to read the input image; in this case, the database is defined in Section 2.2.1 and illustrated in Figure 4;
2. Obtain the inputs for the inference system. The type-1 inference system considers two inputs; these are the gradients calculated with Equations (8) and (9). The inputs are labeled as *Dh* and *Dv*, which represent the gradients in the horizontal and vertical positions, respectively. The two inputs stand for Gaussians membership functions expressed in Equation (3). The *Dh* input is granulated in three MFs with the linguistic variables: “*LowDh*”, “*MiddleDh*”, and “*HighDh*”; the Dv input also has three MFs defined as “*LowDv*”, “*MiddleDv*”, and “*HighDv*”. The parameters are determined according to the gradient values of each image, i.e., considering that the input image is Figure 5, we obtain lower with Equation (14), high using Equation (15), middle with Equation (16), and *σ* values using Equation (17) for the gradients *Dh* and *Dv*.

(14) lowDh=min (Dh),lowDv=min(Dv)

(15) highDh=max(Dh),highDv=max(Dv)

(16) MiddleDh=(lowDh+highDh)/4,MiddleDv=(lowDv+highDv)/4

(17) σDh=highDh /4, σDv=highDv /4

**Figure 6:**
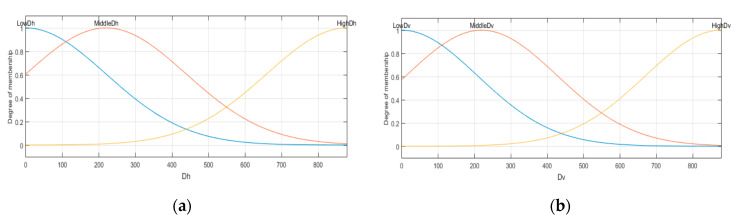
(**a**) Dh input MF; (**b**) Dv input MF.

Considering the gradients in Figure 6, we obtain the values lowDh=0, MiddleDh=219.50, highDh=878, and σDh=219.50 for the input Dh. The parameters for the Gaussian MFs are expressed in Equations (18)–(20) and illustrated in Figure 6a.

(18) $$\mu LowDh\left(x\right)=exp\left[-\frac{1}{2}{\left(\frac{x-0}{219.50}\right)}^{2}\right]$$

(19) $$\mu MiddleDh\left(x\right)=exp\left[-\frac{1}{2}{\left(\frac{x-219.50}{219.50}\right)}^{2}\right]$$

(20) $$\mu HighDh\left(x\right)=exp\left[-\frac{1}{2}{\left(\frac{x-878}{219.50}\right)}^{2}\right]$$

The Gaussian MFs for the Dv input are expressed in Equations (21)–(23) and illustrated in Figure 6b, with the values lowDh=0, MiddleDv=208.50, highDv=834, and σDv=208.50.

(21) $$\mu LowDv\left(x\right)=exp\left[-\frac{1}{2}{\left(\frac{x-0}{208.50}\right)}^{2}\right]$$

(22) $$\mu MiddleDv\left(x\right)=exp\left[-\frac{1}{2}{\left(\frac{x-208.50}{208.50}\right)}^{2}\right]$$

(23) $$\mu HighDv\left(x\right)=exp\left[-\frac{1}{2}{\left(\frac{x-834}{208.50}\right)}^{2}\right]$$

1. Obtain the output. The T1FIS has one output labeled as Edges; this is divided in two linguistic values “*Background*” and “*Edge*”. In this study case, the output (*Edges*) is normalized in a range between −5 and 4.5. The center value for the Background MF is *cBackground* = −5 and for the Edge MF is defined as *cEdge* = 4.5. The σ value for both MFs is calculated with Equation (24);

(24) σoutput=abs(cBackground−cEdge)/2 

The parameters for the output MF (Edges) are expressed in Equations (25) and (26); considering the values of cBackground=−5, cEdge=4.5, and σoutput=4.75.

(25) $$\mu Background\left(x\right)=exp\left[-\frac{1}{2}{\left(\frac{x-\left(-5\right)}{4.75}\right)}^{2}\right]$$

(26) $$\mu Edge\left(x\right)=exp\left[-\frac{1}{2}{\left(\frac{x-4.5}{4.75}\right)}^{2}\right]$$

**Figure 7:**
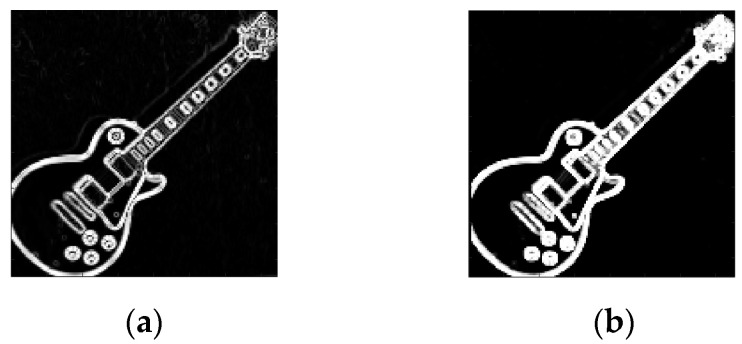
(**a**) Fuzzy Sobel edge-detection output. (**b**) Fuzzy MG output.

2. Mamdani Fuzzy rules. The knowledge base is represented with three fuzzy rules (see Table 2); these are obtained based on expert knowledge. The fuzzy Sobel edge-detection output is illustrated in Figure 7a.

Algorithm 1 summarizes the process for calculating Prewitt and Sobel fuzzy edge-detection.
**Algorithm 1.** Fuzzy edge-detection using Prewitt and Sobel operators.Select the operator to calculate the gradients (Prewitt or Sobel).Case Prewitt: Use kernels *Prewittx* (Equation (4)) and *Prewitty* (Equation (5))Case Sobel: Use kernels *Sobelx* 9 (Equation (6)) and *Sobely* (Equation (7))Read input image fObtain image dimensions (*rows* and *columns*)[*rows*, *columns*] = size(*f*)Calculate the classical gradient corresponding to the fuzzy filter desired[*Dx*, *Dy*] = zeros(*rows*, *columns*) // Generate a zero matrix with the same dimensions as *f* to capture the gradient  for *i* = 0 to rows:   for *j* = 0 to columns:      *Dx[i,j]* = *sum(*kernelx *∗ f [i: i+3, j: j+3])* // Equation (8)      *Dy[i,j]* = *sum(*kernely *∗ f [i: i+3, j: j+3])* // Equation (9)   end for  end forend forGenerate the required fuzzy controller using the fuzzy rules from Table 2 and Table 3.Fuzzify the two input gradients *Dx* and *Dy*, using Gaussian MFs (Equations (18)–(23)).Infer the edges output with the selected controllerDefuzzify the output (*edges*) of the controller.

**Table 3:** Knowledge base with three fuzzy rules for Sobel/Prewitt fuzzy edge-detector.

Inputs		Output	Operator
Dh	Dv	Edges	
HighDh	HighDv	Edge	or
MiddleDh	MiddleDv	Edge	or
LowDh	LowDv	Background	and

**Table 4:** Proposed CNN-I.

Layer/Type	Filters/Neurons	Filter Size
0/Input Convolution + ReLu	40	3 × 3
1/Max Pooling	--	2 × 2
2/Convolution + ReLu	40	3 × 3
3/Max Pooling	--	2 × 2
4/Convolution+ ReLu	60	3 × 3
5/Max Pooling	--	2 × 2
6/Convolution + ReLu	60	3 × 3
7/Max Pooling	--	2 × 2
8/Dropout	--	0.45
9/Flatten	--	--
10/Dense	300	--
11/Dense	250	--
12/Output	6	--

##### Fuzzy Morphological Gradient Edge-Detection

This edge-detector combines the T1 fuzzy set theory and the morphological gradient technique (explained in Section 2.1.3). A fuzzy Mamdani system is implemented that consist of four inputs, one output, and three fuzzy rules; the process is calculated with the following steps.

1. Obtain the four image gradients. After reading the input image, Equations (11) and (12) are used to calculate the image gradients in all directions (*G1*, *G2*, *G3*, and *G4*). Each gradient represents an input in the fuzzy inference system;
2. Define the input MFs and their respective linguistic variables. T1FIS consists of four inputs (*G1*, *G2*, *G3*, and *G4*); each one is separated into three Gaussian membership functions with the linguistic variables “*low*”, “*medium*”, and “*high*”. The parameters for each input MF are adapted depending on the image gray tones; therefore, to calculate the parameters, we obtain the *Low*, *Middle*, and *High* values for each gradient or input using Equations (27)–(30), and these values are used to calculate the σ (Equation (31));
  (27) lowG1=min(G1), lowG2=min(G2),lowG3=min(G3), lowG4=min(G4)
  (28) highG1=max(G1), highG2=max(G2),highG3=max(G3), highG4=max(G4)
  (29) mediumG1=(lowG1+highG1)/2,mediumG2=(lowG2+highG2)/2 
  (30) mediumG3=(lowG3+highG3)/2, mediumG4=(lowG4+highG4)/2
  (31) σG1=highG1/4, σG2=highG2/4,σG3=highG3/4,σG4=highG4/4 

**Figure 8:**
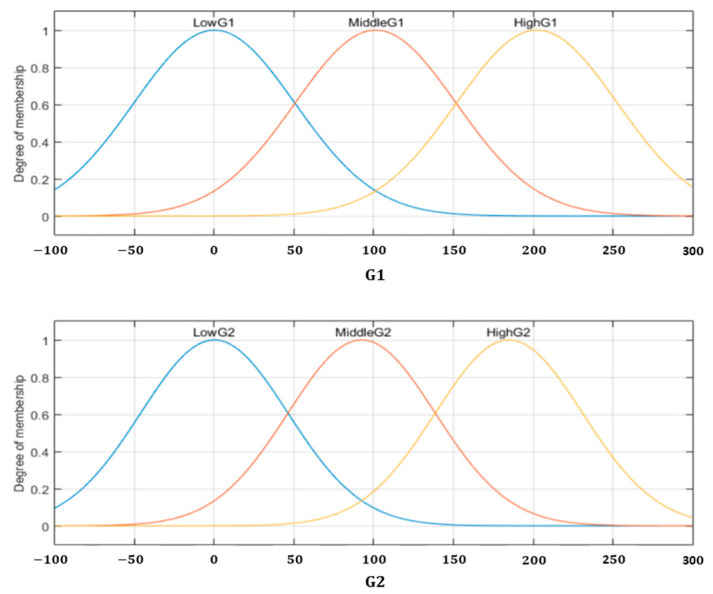
Inputs MFs for the Fuzzy MG.

After calculating the four gradients in Figure 6, we obtain the values of lowG1=0, MiddleG1=219.50, highG1=878, and σG1=219.50 for the input G1; lowG2=0, MiddleG2=219.50, highG2=878, and σG2=219.50 for the input G2; lowG3=0, MiddleG3=219.50, highG3=878, and σG3=219.50 for the input G3; lowG4=0, MiddleG4=219.50, highG4=878, and σG4=219.50 for the input G4. According to these parameters, the Gaussian MFs are denoted by Equations (32)–(43), and Figure 8 illustrates the inputs *G1* and *G2*.

(32) $$\mu LowG1\left(x\right)=exp\left[-\frac{1}{2}{\left(\frac{x-0}{50.5890}\right)}^{2}\right]$$

(33) $$\mu MiddleG1\left(x\right)=exp\left[-\frac{1}{2}{\left(\frac{x-101.1781}{50.5890}\right)}^{2}\right]$$

(34) $$\mu HighG1\left(x\right)=exp\left[-\frac{1}{2}{\left(\frac{x-202.3561}{50.5890}\right)}^{2}\right]$$

(35) $$\mu LowG2\left(x\right)=exp\left[-\frac{1}{2}{\left(\frac{x-0}{46.1912}\right)}^{2}\right]$$

(36) $$\mu MiddleG2\left(x\right)=exp\left[-\frac{1}{2}{\left(\frac{x-92.3824}{46.1912}\right)}^{2}\right]$$

(37) $$\mu HighG2\left(x\right)=exp\left[-\frac{1}{2}{\left(\frac{x-184.7647}{219.50}\right)}^{2}\right]$$

(38) $$\mu LowG3\left(x\right)=exp\left[-\frac{1}{2}{\left(\frac{x-0}{58.1893}\right)}^{2}\right]$$

(39) $$\mu MiddleG3\left(x\right)=exp\left[-\frac{1}{2}{\left(\frac{x-116.3787}{58.1893}\right)}^{2}\right]$$

(40) $$\mu HighG3\left(x\right)=exp\left[-\frac{1}{2}{\left(\frac{x-232.7574}{58.1893}\right)}^{2}\right]$$

(41) $$\mu LowG4\left(x\right)=exp\left[-\frac{1}{2}{\left(\frac{x-0}{44.5884}\right)}^{2}\right]$$

(42) $$\mu MiddleG4\left(x\right)=exp\left[-\frac{1}{2}{\left(\frac{x-89.1768}{44.5884}\right)}^{2}\right]$$

(43) $$\mu HighG4\left(x\right)=exp\left[-\frac{1}{2}{\left(\frac{x-178.3536}{44.5884}\right)}^{2}\right]$$

3. Calculate the output. T1FIS consists of one output (*Edges*), granulated with two linguistic values “*Background*” and “*Edge*”. In the same manner as in the fuzzy Sobel and fuzzy Prewitt approaches, the output edges matrix is normalized in a range between −5 and 4.5; the center value for the Background MF is cBackground=−5 and the Edge MF is cEdge=4.5. The σ value for both MFs is calculated with Equation (44);
  (44) σoutput=abs(cBackground−cEdge)/2

**Figure 9:**
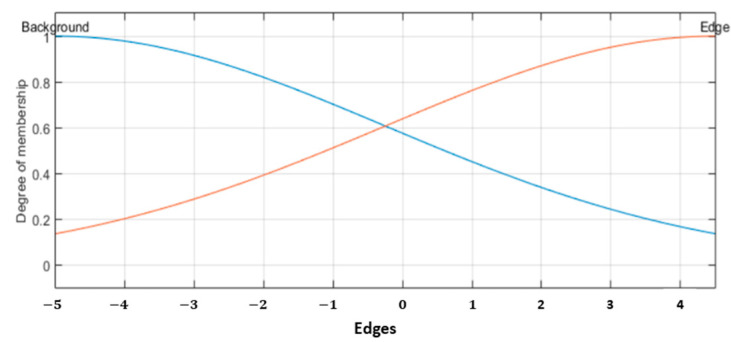
Output MF for the Fuzzy MG.

The parameters for the output MF (Edges) are expressed in Equations (45) and (46) and illustrated in Figure 9, considering the values of cBackground=−5, cEdge=4.5, and σoutput=4.75.

(45) $$\mu Background\left(x\right)=exp\left[-\frac{1}{2}{\left(\frac{x-\left(-5\right)}{4.75}\right)}^{2}\right]$$

(46) $$\mu Edge\left(x\right)=exp\left[-\frac{1}{2}{\left(\frac{x-4.5}{4.75}\right)}^{2}\right]$$

4. Mamdani Fuzzy rules. The knowledge base is represented with three fuzzy rules (Table 2). The fuzzy MG edge-detection output is illustrated in Figure 7b.

#### 2.2.3. CNN Training and Validation

For this research, we experimented with three architectures: two proposed models of convolutional neural networks and a pre trained VGG16 model [49]. The proposed models were crafted with the ideology of achieving high levels of accuracy without consuming large amounts of computing resources, such as the VGG16 or Inception models [50], which are very large models trained for multiple categories. In this case, the model CNN-I (architecture overview in Table 4) consists of four convolution layers that include the rectified linear unit activation (ReLu) and maximum pooling in between each convolution layer, a dropout of 45%, two dense layers, and the output layer. The CNN-II model (architecture overview in Table 5) is a variant of the first model where the neurons in the convolutional layers are increased to extract as much information as possible from the filter; as well as CNN-I, each of these convolution layers includes a ReLu activation, and each convolution is followed by a maximum pooling layer with the same filter size. To compensate for overfitting the number of neurons used, the dropout layer is increased to turn off 75% of the neurons before passing to a single, but significantly larger, fully connected layer and finally going to the output.

The hyperparameters used to train the models were determined by experimentation. After many trials, the best results were obtained using the following parameters:

- Epochs: 30;
- Loss function: Sparce categorical cross entropy;
- Training algorithm: Adam (default settings in Keras API);
  ○ Epsilon: 1 × 10^−7^;
  ○ Beta 1: 0.9;
  ○ Beta 2: 0.999;
  ○ Learning rate: 0.001;
- Batch size: 60.

In the case of the pre-trained model, we used a VGG16 from the TensorFlow library, using the weights from the ImageNet dataset [51]. The weights were updated by performing transfer learning. This was done by removing the last (output) layer of the model and freezing the training parameters of the other layers. A new single classification layer was added with six neurons (one for each class) and we retrained the model with the following parameters:

- Epochs: 15;
- Loss function: Sparce categorical cross entropy;
- Training algorithm: Adam (default settings in Keras API);
  ○ Epsilon: 1 × 10^−7^;
  ○ Beta 1: 0.9;
  ○ Beta 2: 0.999;
  ○ Learning rate: 0.001;
- Batch size: 80.

For the training of these models, we performed K-fold cross-validation; in this process, division of the dataset was performed to create multiple training and test sets denominated as folds. This process was performed to evaluate the model with the entire dataset. Fuzzy preprocessing showed an overall performance improvement over the non-preprocessed images (grayscale and RGB).

## 3. Results

To compare the efficiency of the proposed models, we implemented them in Python, using the TensorFlow framework with the Keras API. Summaries of the results of the 30 experiments performed for each preprocessing approach for CNN-I and CNN-II are shown in Table 6 and Table 7, respectively, and the results for the VGG16 model are displayed in Table 8. Fuzzy preprocessing showed an overall performance improvement against the grayscale images and the classic edge-detection methodologies. The experiments were performed on a laptop with the following specifications: CPU Intel core i7-11800H, 32 GB DDR4 RAM at 3200 MHz, and an Nvidia RTX 3080 Laptop GPU with 16 GB of video memory.

**Figure 10:**
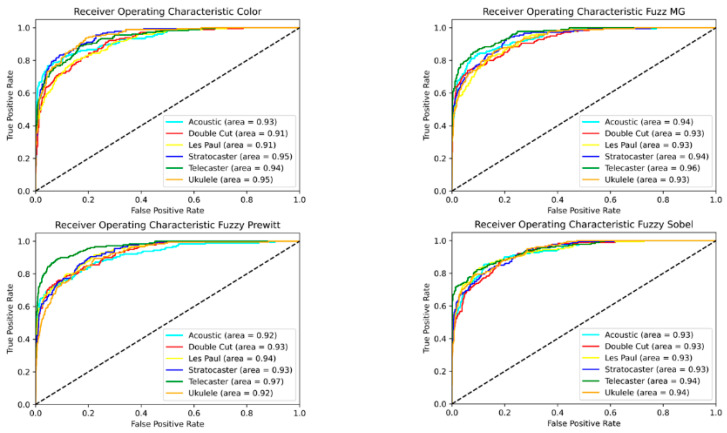
ROC curve for CNN-I.

**Figure 11:**
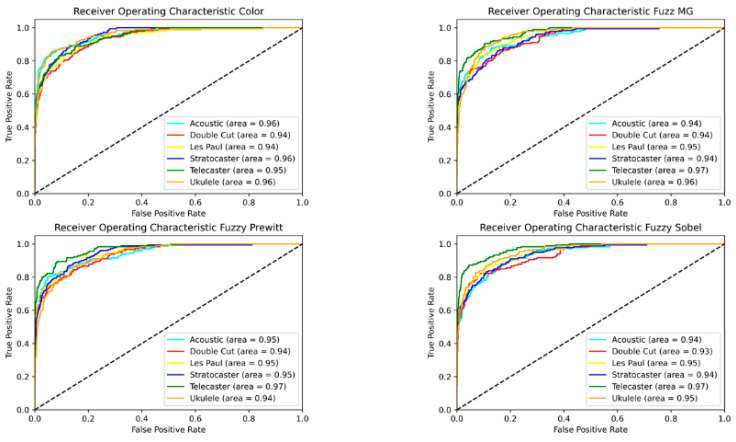
ROC curve for CNN-II.

To evaluate the performance between the fuzzy preprocessing techniques and the color images, we calculated a ROC curve to have a visual representation of the performance of each class. Curves were calculated for both proposed models, as shown in Figure 10 for CNN-I and Figure 11 for CNN-II, each time with the best instance of the models trained.

**Figure 12:**
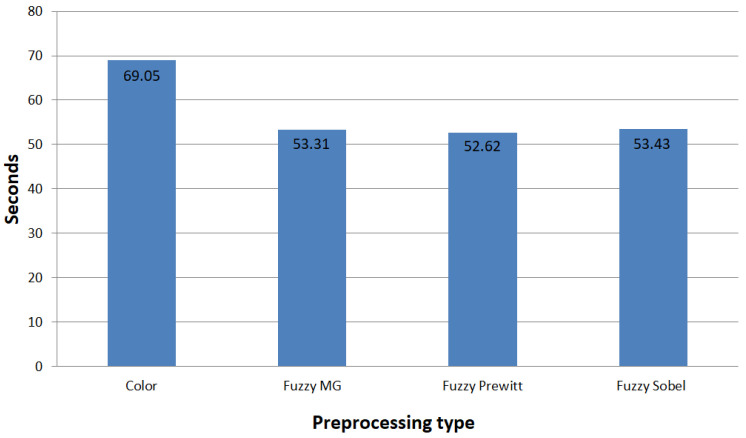
Average training time per model in seconds for CNN-I.

**Figure 13:**
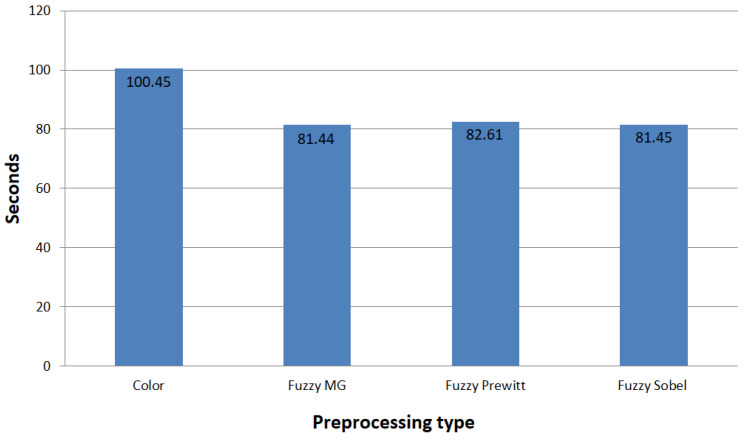
Average training time per model in seconds for CNN-II.

To expand the results of the ROC graph where the RGB images presented a slightly lower accuracy when compared to some of the fuzzy detectors, we calculated the average training time for each model. Figure 12 contains the average training time per model in seconds for CNN-I, and Figure 13 presents the averages for CNN-II.

### Statistical Test between Classic and Fuzzy Preprocessing Techniques

To verify the existence of significant evidence of the performance gain obtained with the different fuzzy preprocessing techniques, a Z-test statistical analysis was applied to compare the fuzzy preprocessing approaches (fuzzy MG edge-detection, fuzzy Sobel edge-detection, and fuzzy Prewitt) against the raw images (only resizing the images to fit the model). The tests for each preprocessing methodology were made independent for each model. The Z-test was a right-tailed test; the parameters used for the tests were the following:

- Right-tailed test;
- α=0.05 (95% confidence level, rejection zone at $z_{c}=1.96$);
- n=30;
- $H_{0}$: Fuzzy preprocessing approach $\left(\mu_{1}\right)$ offers less or equal accuracy than the raw images $\left(\mu_{2}\right)$. $H_{0}$: $\mu_{1}\leq \;\mu_{2}$;
- $H_{a}$: Fuzzy preprocessing approach $\left(\mu_{1}\right)$ offers more accuracy than the raw images $\left(\mu_{2}\right)$. $H_{a}$: $\mu_{1}>\mu_{2}$ (affirmation).

The results of the null hypothesis test for CNN-I are shown in Table 9. The $\bar{x_{1}}\;\mathrm{and}\;\sigma_{1}$ variables represent the mean and standard deviation, respectively, for the fuzzy preprocessing approach; $\bar{x_{2}}\;\mathrm{and}\;\sigma_{2}$ represent the mean and standard deviation, respectively, for raw images and grayscale.

The results for the Z-test for the CNN-II model are then shown in Table 10. 

## 4. Discussion

As demonstrated in the literature review, in addition to the results obtained, implementing fuzzy image preprocessing techniques before feeding the images into a convolutional neural network has proven to be beneficial in most instances for improving the accuracy of the model. As shown in Table 6 and Table 7, the maximum accuracy was obtained with fuzzy Sobel and fuzzy Prewitt for CNN-I, with a value of 71.76% in both cases after performing the 30 experiments; in the case of the CNN-II, fuzzy Sobel achieved the best model performance, obtaining the maximum accuracy of 75.18%. These are competitive results against RGB images, where the latter do not require preprocessing but need more computational resources and time to train the model.

When reviewing the classical edge-detection methodologies for CNN-I, we can note that Sobel and MG preprocessing tended to decrease the accuracy, with an average of 53.07% for MG and 64.72% for Sobel. With Prewitt, we obtained the best average for the model with 67.63%, even better than the RGB images, which gave a result of 66.87%. The average for the fuzzy preprocessing offered a slight improvement of almost 1% when compared to the RGB images in all instances. The results obtained with CNN-II also followed a tendency where the classic Sobel and MG offered a decrease in the average of almost 15% for MG and 4% for Sobel. We also noted comparable averages with the fuzzy approaches and with Prewitt preprocessing; all four options were comparable to RGB images with a delta of ±1%.

In the case of CNN-II, even though the model had an overall accuracy improvement, when compared to CNN-I, we can note a similar pattern to CNN-I where classical edge-detection methodologies suffered an accuracy loss compared to grayscale images, though applying fuzzy filters allowed us to surpass the grayscale approach. In this case, the performance loss with MG edge-detection was more significant, with a 14.34% lower accuracy. In the case of the Sobel edge-detection, we had a 2.41% accuracy loss on average. Fuzzy Prewitt reported a 1.08% accuracy improvement when compared to the grayscale preprocessing, and a 0.18% accuracy improvement when compared to the color images.

The results obtained with the pre-trained VGG16 model, denoted in Table 8, were not as impressive as those for the specialized model without preprocessing, especially when compared to CNN-II with fuzzy Sobel, which gave over a 1.9% accuracy improvement with the best models. This alternative could represent a decent out-of-the-box implementation, without taking into consideration the training times needed, because the model utilizes 224 × 224 px RGB images instead of the 150 × 150 px grayscale used for fuzzy Sobel.

The results can be validated with the ROC curves shown in Figure 10 and Figure 11, which show the sensitivity and specificity of the models. We can observe that each class in every model has a similar performance, where the models are performing a decent separation of the classes. The models present a similar level of area under the curve (AOC) due to the relatively small differences between them; we found no more than a 2% difference between preprocessing methodologies and no more than a 5% difference between the best CNN-I models compared to the best of CNN-II models.

The ROC curves shown in Figure 10 and Figure 11 demonstrate similar behavior in terms of accuracy between the fuzzy preprocessing and the color (RGB) images. To evaluate the effectiveness of the proposed methodologies in both architectures, we performed a time analysis by training 30 independent models, to evaluate the average training time, and we calculated the average preprocessing time for the images (Figure 12 and Figure 13). With CNN-I, we noted a reduction by an average of 23% in the training time with the fuzzy preprocessing. In the case of CNN-II, we had a reduction in time of 18.5% with the more complex architecture. In both instances, we managed to achieve similar accuracy rates with a significant reduction in training time. These reductions can be significant in similar applications in which the models need to be trained with more information.

The models trained with a preprocessing step showed a clear advantage in training time when compared to the color images, due to the utilization of a single layer, instead of the three layers used in RGB images. The trade-off between the optimization in training times and doubling the preprocessing step time is directly reflected in GPU usage, which normally represents the highest upfront cost when compared to CPU usage, in the development of these types of systems.

The results of the statistical analysis performed, demonstrated in Table 9 and Table 10, show that for CNN-I, the comparison between the utilization of fuzzy preprocessing against RGB images revealed a significant difference with the fuzzy Prewitt application, with a Z-score of 2.3. In the other two instances, the fuzzy filters presented better accuracy, but the threshold was not reached. On the other hand, when comparing the fuzzy filters against the grayscale images, we can note that all the Z-scores surpassed the 1.96 threshold, therefore, providing enough evidence to accept our alternative hypothesis, making the fuzzy preprocessing step a compelling alternative to improve accuracy. The results obtained for CNN-II demonstrated that there is sufficient evidence to validate the usage of a fuzzy filter (Prewitt) when compared to the grayscale model. The main support for this was the lack of margin between the results, where the margins of improvement in precision were not as tangible as for CNN-I, which had less capacity to extract characteristics from which it benefitted on multiple occasions with the use of fuzzy edge-detectors.

## 5. Conclusions and Future Work

The experimentation and statistical analysis showed that implementing fuzzy edge-detection for images before feeding them into the convolutional layers in a DNN (in some instances) can significantly improve the accuracy of the trained models, while reducing training times, especially in model CNN-I, where less filters are used for feature extraction. On other hand, model CNN-II, with more filters per layer, demonstrated improved accuracy, but no significant evidence was found to validate the usage of preprocessing layers.

When investigating the pre-trained VGG16 model, a 2% reduction in accuracy was found compared to CNN-2 with the RGB images, making it a poor alternative, especially considering the model size and training times.

We believe that the main limiting factors that affected our results were the complexity of our training information, with vast differences between images in the same category of the dataset, as well as similarities between the classes. In the future, we would like to improve the dataset and develop a more curated version that does not include the huge variation in the current version, and we would like to implement the proposed preprocessing methodology and model to benchmark datasets, to compare the efficiency against state-of-the-art models.

## Figures and Tables

**Table 1:** Guitar database description.

Name	Description
Total images	5400
Training images	4320
Test images	1080
Image size	150 × 150
Data base format	JPG

**Table 2:** Fuzzy rules base for fuzzy MG edge-detector.

Inputs				Output	Operator
G1	G2	G3	G4	Edges	
HighG1	HighG2	HighG3	HighG4	Edge	or
MiddleG1	MiddleG2	MiddleG3	MiddleG4	Edge	or
LowG1	LowG2	LowG3	LowG4	Background	and

**Table 5:** Proposed CNN-II.

Layer/Type	Filters/Neurons	Filter Size
0/Input Convolution + ReLu	64	3 × 3
1/Max Pooling	--	2 × 2
2/Convolution + ReLu	64	3 × 3
3/Max Pooling	--	2 × 2
4/Convolution + ReLu	128	3 × 3
5/Max Pooling	--	2 × 2
6/Convolution + ReLu	128	3 × 3
7/Max Pooling	--	2 × 2
8/Dropout	--	0.75
9/Flatten	--	--
10/Dense	512	--
11/Output	6	--

**Table 6:** Results of the CNN-I model.

Preprocessing	Min.	Max.	Average	Standard Deviation
RGB	0.6296	0.7127	0.6687	0.0141
Grayscale	0.6135	0.7037	0.6601	0.0199
MG	0.4870	0.5759	0.5307	0.0177
Prewitt	0.6305	0.7129	0.6763	0.0163
Sobel	0.5894	0.6956	0.6472	0.0282
Fuzzy MG	0.6315	0.7167	0.6708	0.0186
Fuzzy Prewitt	0.6380	0.7176	0.6775	0.0151
Fuzzy Sobel	0.6380	0.7176	0.6751	0.0196

**Table 7:** Results of the CNN-II model.

Preprocessing	Min.	Max.	Average	Standard Deviation
RGB	0.6796	0.7491	0.7121	0.0137
Grayscale	0.6654	0.7463	0.7031	0.0176
MG	0.5102	0.5991	0.5597	0.0177
Prewitt	0.6675	0.7481	0.7130	0.0139
Sobel	0.6712	0.6898	0.6789	0.01794
Fuzzy MG	0.6657	0.7444	0.7041	0.0153
Fuzzy Prewitt	0.6824	0.7481	0.7157	0.0145
Fuzzy Sobel	0.1667	0.7518	0.7030	0.0479

**Table 8:** Results of the VGG16.

Preprocessing	Min.	Max.	Average	Standard Deviation
RGB	0.6398	0.7356	0.6908	0.0203

**Table 9:** Hypothesis test results for CNN-I model.

Preprocessing	$\bar{x_{1}}$	$\sigma_{1}$	$\bar{x_{2}}$	$\sigma_{2}$	Z-Score
Fuzzy Sobel vs. RGB	0.6708	0.0186	0.6687	0.0141	1.4347
Fuzzy Prewitt vs. RGB	0.6775	0.0151	0.6687	0.0141	2.3336
Fuzzy MG vs. RGB	0.6751	0.0196	0.6687	0.0141	0.4892
Fuzzy Sobel vs. grayscale	0.6708	0.0186	0.6601	0.0199	2.9244
Fuzzy Prewitt vs. grayscale	0.6775	0.0151	0.6601	0.0199	3.8144
Fuzzy MG vs. grayscale	0.6751	0.0196	0.6601	0.0199	2.1474

**Table 10:** Hypothesis test results for CNN-II model.

Preprocessing	$\bar{x_{1}}$	$\sigma_{1}$	$\bar{x_{2}}$	$\sigma_{2}$	Z-Score
Fuzzy Sobel vs. color	0.7041	0.0153	0.7121	0.0137	−0.9961
Fuzzy Prewitt vs. color	0.7157	0.0145	0.7121	0.0137	1.0065
Fuzzy MG vs. color	0.7030	0.0479	0.7121	0.0137	−2.1186
Fuzzy Sobel vs. grayscale	0.7041	0.0153	0.7031	0.0176	−0.006
Fuzzy Prewitt vs. grayscale	0.7157	0.0145	0.7031	0.0176	3.0494
Fuzzy MG vs. grayscale	0.7030	0.0479	0.7031	0.0176	0.2462

## Data Availability

Not applicable.
